# Sexual function and Dyadic adjustment in women with urinary incontinence

**DOI:** 10.12669/pjms.35.2.296

**Published:** 2019

**Authors:** Sureyya Gumussoy, Oya Kavlak, Sevgul Donmez

**Affiliations:** 1 *Dr. Sureyya Gumussoy, Ege University Ataturk Health Care Vocational School, Izmir, Turkey*; 2 *Professor Oya Kavlak, Ege University Ataturk Health Care Vocational School, Izmir, Turkey*; 3 *Dr. Sevgul Donmez, Faculty of Health Sciences, Mugla University, Mugla, Turkey*

**Keywords:** KEY WORDS: Dyadic adjustment, Sexual function, Urinary incontinence

## Abstract

**Objective:**

This study was planned to evaluate the effects of urinary incontinence (UI) on sexual function (SF) and dyadic adjustment.

**Methods:**

The study was conducted with 203 women with urinary incontinence. This study was conducted at Urogynecology Outpatient Clinic of our hospital between September 2017 and February 2018. Data were collected using the Dyadic Adjustment Scale (DAS), and “Female Sexual Function Index (FSFI).

**Results:**

The incidence of sexual dysfunction (SD) was higher in the patients who were in advanced age, had a husband older than them, entered menopause, had lower levels of education, had the higher frequency of UI and changed pads more frequently, and these patients had lower DAS scores.

**Conclusion:**

It was determined that the majority of the participating women with UI experienced SD and that those with SD had lower DAS scores.

## INTRODUCTION

The International Continence Society described UI as the involuntary loss of urine that can be objectively assessed and causes hygienic and social problems in an individual. UI is a widespread problem that affects individuals and their families not only from physical, psychological and social aspects but also from a financial aspect. It is known that more than 200 million people in the world have incontinence problems and that the majority of them are women.^1^ The prevalence of UI that can affect women of all ages ranges from 10% to 30% in women aged 15-64 years and from 17% to 55% in elderly women. Despite its high prevalence rate, UI is an issue often not voiced, and women are reluctant to talk or to receive help on this matter. Most women consider UI as a social problem and taboo rather than a medical issue, abstain from talking about the problem, and often visit a physician at least a year after the problem begins.^2^ One of the most important problems affecting the woman with UI and her partner is sexual dysfunction.^3^ That is because UI affects sexual life negatively due to problems faced during the sexual intercourse such as urinary leakage, embarrassment, wetness and odor, and thus has an important role in the etiology of sexual dysfunctions. In several studies conducted on the issue, it has been determined that UI is associated with lack of libido, vaginal dryness, dyspareunia, decreased sexual desire, sexual arousal disorder and orgasm problems.^4^ Due to these problems, the woman’s relationship with her partner deteriorates and harmony between the couple is affected adversely. In the literature, although there is not a study investigating the effect of UI on dyadic adjustment, it is thought that UI may adversely affect dyadic adjustment because of its negative effects on women’s sexual functions. Women with UI try different ways to hide the problem from their partners or they try to avoid sexual intercourse.

Therefore, it should also be kept in mind that the sexual problems of partners indirectly affect each other’s sexual functions.^5^ Marital adjustment is a multivariable phenomenon, including concepts such as how the woman and man perceive their relationships, how well their expectations are met, whether they are emotionally satisfied and whether they enjoy the sexual intercourse.^6^,^7^ Today, it has been determined that whether mild or strong, people’s problems related to sexual functioning, and satisfaction from sexual life are associated with their dissatisfaction with their relationships, and that the less the happiness and the fewer the shared activities in marriage, the greater the likelihood of decreased sexual activity and of the alienation of affection between spouses.^8^ This study was planned to evaluate the effects of urinary incontinence (UI) on sexual function (SF) and dyadic adjustment.

## METHODS

This descriptive and correlational study was carried out with 203 women with UI admitted to Ege University Hospital Urogynecology Outpatient Clinic between September 2017 and February 2018 due to UI. The sample was selected using the simple random improbable sampling method. The study included women who were sexually active, were diagnosed with UI, were able to communicate, and did not have a psychiatric disease that required significant medical intervention within the last 1 year, or had no urinary or genital system infection and volunteered to participate in the study. Women who were illiterate were excluded from the study. Of the patients in the study population, 83.0% were reached.

The study data were collected using the Sociodemographic Characteristics Questionnaire, DAS and FSFI. The women included in the sample were asked to fill in the data collection forms individually, after they were informed about the purpose of the study and told that participation was completely voluntary, and that they could withdraw from the study at any time.

### Sociodemographic Characteristics Questionnaire

The Questionnaire developed by the researchers in the light of the literature includes 20 items questioning the socio-demographic characteristics of women diagnosed with UI and their characteristics related to menopause and UI.^4^,^5^

### Dyadic Adjustment Scale

The scale consists of 32 items. The validity and reliability study of the Turkish version of the scale was conducted by Fisiloglu and Demir. The lowest and highest possible scores to be obtained from the scale are 0 and 151 respectively. The higher the score obtained from the scale is, the better the quality of the relationship is.^9^

### Female Sexual Function Index

The FSFI which has 19 items. With the scale, sexual problems or functions in the last four weeks are assessed. The reliability and validity study of the Turkish version of the scale was carried out by Aygin and Arslan in 2005.^10^

To analyze the data, Kruskal Wallis and Mann Whitney U tests were used. The results were evaluated at a 95% confidence interval and a significance level of p <0.05. We obtained the approval of the scientific ethics committee at Nursing Faculty. To carry out the study, written permission was obtained from the hospital and the participants.

## RESULTS

FSFI, DAS mean score was 18.29±5.97 and 97.65±16.82. The analysis revealed a weak, statistically significant positive relationship between the mean scores the participants obtained from the FSFI and DAS (Table-I). While 91.1% of the women participating in the study experienced sexual dysfunction, 8.9% did not. The comparison of the participants’ DAS scores by their socio-demographic characteristics and SD revealed a statistically significant relationship between their DAS scores and variables such as age, educational status, employment status, and income status, place of residence, entering menopause and SD (Table-II). The Comparison of the Mean Scores Obtained by the Participants from the FSFI and DAS in Terms of their UI status are given in Table-III.

**Table-I:** Distribution of the Mean FSFI and DAS Scores and Correlation between the Mean Scores.

Items	Mean ± SD	Min-Max Score of Them	DAS	

FSFI			r	p
Desire	2.83±1.07	1.20-4.80	0.322	0.001*
Arousal	2.95±1.12	0.90-5.70	0.290	0.001*
Lubrication	3.17±1.13	0.90-6.00	0.342	0.001*
Orgasm	3.12±1.15	1.20-6.00	0.338	0.001*
Satisfaction	3.05±1.21	1.20-6.00	0.336	0.001*
Pain	3.14±1.28	1.20-6.00	0.341	0.001*

**Total**	18.29±5.97	7.20-33.90	FSFI	

*DAS*			*r*	*p*

Dyadic Consensus	49.00±8.79	27-70	0.398	0.001*
Dyadic Satisfaction	32.62±5.99	20-47	0.246	0.001*
Dyadic Cohesion	7.37±1.91	1-12	0.461	0.001*
Affectional Expression	15.08±3.89	1-25	0.310	0.001*

**Total**	97.65±16.82	55-136	0.384	0.001*

* p<0.05

**Table-II:** The FSFI and DAS Mean Scores by their Sociodemographic Characteristics.

	Female SD									

Variables	Yes		No		Total		X²	P	DAS Scores	

	n	%	n	%	n	%				
***Age (years)***										
30-39	28	15.1	6	33.3	34	16.7			98.08±15.5	
40-49	57	30.8	11	61.1	68	33.5	15.537	0.001*	101.28±16.4	K=2.328 p=0.127
50 and older	100	54.1	1	5.6	101	49.8			95.06±17.1	
***Spouse age (years)***										
30-39	12	6.5	3	16.7	15	7.4			97.93±15.6	
40-49	42	22.7	11	61.1	53	26.1	17.906	0.001*	99.83±16.6	K=1.822 p=0.177
50 and older	131	70.8	4	22.2	135	66.5			96.77±17.0	
***Educational***										
Elementary	107	57.8	3	16.7	110	54.2			96.80±17.2	
High school	62	33.6	9	50.0	71	35.0	15.629	0.001*	96.74±16.5	K=5.067 p=0.079
University or higher	16	8.6	6	33.3	22	10.8			104.82±14.1	
***Spouse educational***										
Elementary	64	34.6	1	5.6	65	32.0			94.29±18.0	
High school	105	56.8	12	66.7	117	57.6	10.402	0.006*	98.9±15.7	K=4.048 p=0.132
University or higher	16	8.6	5	27.8	21	10.4			100.81±17.8	
***Occupation***										
Yes	31	27.6	11	61.1	62	30.5			98.01±17.7	
Not working	134	72.4	7	38.9	141	69.5	8.701	0.003*	97.49±16.4	U=4230.5 p=0.715
***Perception of income***										
Insufficient	108	58.4	7	38.9	115	56.7			95.68±17.7	
Sufficient	77	41.6	11	61.1	88	43.3	13.814	0.001*	100.02±15.2	U=4240.0 p=0.048
***Living region***										
Village	14	7.5	0	0.0	14	6.9			84.64±12.3	
Town	73	39.5	3	16.7	76	37.4	6.355	0.042*	92.53±13.5	K=28.532 p=0.001*
Province	98	53.0	15	83.3	113	55.7			102.71±17.4	
***Duration of marriage***										
≤10 years	15	8.1	3	16.7	18	8.9			95.44±16.1	
≥11 years	170	91.9	15	83.3	185	9.11	1.487	0.223	98.87±16.8	U=1548.0 p=0.623
***Status of menopause***										
Yes	94	50.8	2	11.1	96	47.3			95.15±16.4	
No	91	49.2	16	88.9	107	52.7	10.372	0.001*	99.89±16.9	U=4290.0 p=0.043*

* p<0.05

**Table-III:** The FSFI and DAS Mean Scores in Terms of their UI Status.

	Female SD									

Variables	Yes		No		Total		X²	P	DAS Scores	

	n	%	n	%	n	%				
***Time of UI(years)***										
≤5	124	67.0	17	94.4	141	69.5			102.73±16.6	
6 and above	61	33.0	1	5.6	62	30.5	5.813	0.016*	86.57±11.2	U=2122.5 p=0.001*
***Used pads when they leaked urine***										
Yes	106	57.3	5	27.8	111	54.7			89.36±13.1	
No	79	42.7	13	72.2	92	45.3	5.768	0.016*	109.21±15.2	U=2432.5 p=0.001*
***Frequencies of UI***										
Once a day	36	19.5	1	5.6	37	18.2			98.88±17.3	
More than once a day	80	43.2	8	11.1	82	40.4	18.146	0.003*	88.24±12.2	
Once a week	28	15.1	8	44.4	36	17.7			103.75±21.3	K=30.611 p=0.001*
More than once a week	22	11.9	2	11.1	24	11.8			103.89±15.2	
A few times a month	15	8.1	4	22.2	19	9.4			96.20±22.2	
Rarely	4	2.2	1	5.6	5	2.5			121.33±0.57	

## DISCUSSION

UI has become a serious problem affecting women’s quality of life globally. One of the most important parameters that constitute the quality of life of a woman is her sexual life.^3^,^6^ It has been reported that due to loss of self-esteem, psychological pressure and dyspareunia caused by contact dermatitis due to urinary contact, UI leads to SD in 25-50% of women with the problem.^11^ Similarly, in the present study, the majority of the participating women with UI suffered from sexual dysfunction. In another study, 32-68% of the women with UI experienced sexual dysfunction.^12^ In a review investigating the sexuality in women with UI, it is stated that more than 50% of the women with UI suffer from sexual dysfunction.^13^ In the literature, the high prevalence of SD in women with incontinence is associated with the fact that they avoid receiving medical help because of embarrassment, hesitation, low expectation of treatment and perception that SD is a part of aging and the female sex.^14^ It is known that in Turkey, while most clinicians generally focus on the primary treatment of the current pelvic disease and neglect to question sexual function, women refrain from asking questions on this matter before treatment.^12^

In the study, there was a weak, statistically significant positive relationship between the mean scores the participants obtained from the FSFI and DAS. In the literature, it is stated that one of the most important psychosocial effects of UI is observed in marital relations.^8^ UI adversely affected the lives of women with the problem and their relationship with their partners. In the another study, 38% of the women and 32% of the men stated that the women’s urinary problems had a negative impact on their relationship.^15^,^16^ Kizilkaya Beji N et al.’s study indicated that that 50% of the patients hide their urine leakage problems from their husbands, 19% went to the toilet to urinate before sexual intercourse, and more importantly, 28% avoided having sexual intercourse.^17^ A woman with UI loses her self-esteem, feels embarrassed, gets less and less pleasure from sexual intercourse and eventually refrains from having sexual intercourse. As a consequence of all these, the institution of marriage and the relationship between the spouses are affected negatively.^3^

It is thought that not only UI but also advanced age and menopause lead to sexual dysfunction. In the current study we found that the incidence of SD increased and the mean DAS scores decreased after the women entered menopause or their husbands aged. Lukacz ES et al. suggested that women’s sexuality was negatively affected by menopause and age.^18^ In another study conducted to compare women with UI and women without UI, the rates of SD were higher in menopausal women, advanced age women and women whose husbands were older than them^19^ which is thought to result from problems such as the decrease in sexual arousal, difficulty in achieving orgasm or being sexually unsatisfied all of which are fueled by UI.^20^,^21^

In the present study, the incidence of SD increased and the mean DAS scores decreased as the level of education decreased. There was an inverse correlation between the participants’ education levels and sexual problems in Laumann EO et al.’s study.^22^

Because of the many negative effects of UI on sexual function, the woman’s relationship with her husband deteriorates and the family structure is negatively affected.^23^ Similarly, in the present study, of the participating women, those who had UI or changed pads more frequently suffered from SD more and obtained statistically significantly lower DAS scores. Guducu N et al. found that sexual functions of the women worsened as the number of the pads they changed and the frequency of urinary leakage increased.^24^ Thus, it can be concluded that the physiological complaints of the woman and her partner due to the severity of the UI may increase the degree of SD and affect their relations adversely. The impact of UI on the family health of women brings the importance of multidisciplinary clinical evaluation of individuals and couples and planning of care strategies to the forefront.^25^

## CONCLUSION

It was determined that the majority of the participants with UI suffered from SD and those with SD had lower dyadic adjustment scores. In addition, the incidence of sexual dysfunction was higher in the patients who were in advanced age, entered menopause, had lower levels of education, had the higher frequency of UI and wore pads more, and these patients had lower mean DAS scores. Due to these adverse effects of UI on family and sexual life, it should be regarded as a serious health problem regardless of at what period of life it arises and should be treated with appropriate methods such as conservative treatment (e.g. bladder training, dieting, creating a toilet program, habit training, encouraging urination), pelvic floor muscle exercises, administration of alpha adrenergic drugs, antidepressants and anticholinergics, and surgical methods. In addition, it is assumed that determination of emotions, thoughts and perceptions of women with UI about sexual function and their harmony with their partners may lead to changes in approaches towards the problems of urinary incontinence both in the community and in clinics, and that the awareness of both the patient population seeking help and the healthcare personnel and thus the success rate in UI management will increase as the awareness of urinary incontinence and treatment is increased. In addition, how the emotional health and quality of life of the partners of women with UI are affected has been relatively neglected. Therefore, there is a need for prospective studies investigating effects of UI on sexual function and family life in larger samples.

### Author`s Contribution

**SG, OK and SD** conceived, designed and did statistical analysis & editing of manuscript.

**SG and SD** did data collection and manuscript writing.

**SG, OK and SD** did review and final approval of manuscript.
